# Anti-SARS-CoV-2 IgM Antibody Levels Measured by an In-House ELISA in a Convalescent Latin Population Persist over Time and Exhibit Neutralizing Capacity to Several Variants of Concern

**DOI:** 10.3390/diagnostics14192209

**Published:** 2024-10-03

**Authors:** Ana M. Espino, Albersy Armina-Rodriguez, Paola Cardona, Carlimar Ocasio-Malavé, Laura Alvarez, Carlos A. Sariol

**Affiliations:** 1 Department of Microbiology and Medical Zoology, University of Puerto Rico-Medical Sciences Campus, San Juan, PR 00936, USA; albersy.armina@upr.edu (A.A.-R.); carlimar.ocasio@upr.edu (C.O.-M.); laura.alvarez@upr.edu (L.A.); 2 School of Health Professions, University of Puerto Rico-Medical Sciences Campus, San Juan, PR 00936, USA; paola.cardona@upr.edu; 3 Unit of Comparative Medicine, Department of Medicine, University of Puerto Rico-Medical Sciences Campus, San Juan, PR 00936, USA

**Keywords:** COVID-19, IgM, indirect ELISA, neutralizing antibody, Latin, Puerto Rico

## Abstract

Background: The coronavirus, SARS-CoV-2, is the causative agent for COVID-19, first registered in Wuhan, China and responsible for more than 6 million deaths worldwide. Currently, RT-PCR is the gold-standard method for diagnosing COVID-19. However, serological tests are needed for screening acute disease diagnosis and screening large populations during the COVID-19 outbreak. Objectives: Herein, we described the development and validation of an in-house enzyme-linked immunosorbent assay (ELISA) for detecting the levels of anti-spike-1-RBD IgM antibody (CovIgM-ELISA) in well-defined serum/plasma panel for screening and identifying subjects infected with SARS-CoV-2 in a Latin population. Method: In-house CovIgM-ELISA has the format of an indirect ELISA. It was optimized by checkerboard titration using recombinant SARS-CoV-2 spike-S1-RBD protein as an antigen. Results: We found that, compared to the RT-PCR as the standard method, the in-house CovIgM-ELISA displayed sensitivities of 96.15% and 93.22% for samples collected up to 30 or 60 days after infection, respectively, as well as 95.59% specificity with 97.3% accuracy. The agreement kappa value (*κ*) of our CovIgM-ELISA was substantial when compared to RT-PCR (κ = 0.873) and the anti-SARS-CoV-2 IgM ELISA (InBios Int) (κ = 0.684). The IgM levels detected in the population positively correlated with the neutralizing activity against the wild-type, Alpha and Delta variants of concern, but failed to neutralize Omicron. Conclusions: These data indicate that our in-house CovIgM-ELISA is a compatible performing assay for the detection of SARS-CoV-2 infection.

## 1. Introduction

SARS-CoV-2, the Coronavirus causative of severe acute respiratory syndrome, was identified in Wuhan, China in 2019, and spread quickly to several other countries. On 30 January 2020, the disease caused by this new Coronavirus, designated as COVID-19, was declared by the World Health Organization (WHO) as a Public Health Emergency of International Concern [1]. Thereafter, it was formally declared as pandemic in March 2020. By 15 November 2021, SARS-CoV-2 has been responsible for more than 500 million infections and over 5 million deaths worldwide [2].

The laboratory diagnosis of SARS-CoV-2 infections has been primarily based on detection of viral RNA via reverse transcription polymerase chain reaction (RT-PCR), which is considered the gold-standard detection method for COVID-19. While RT-PCR is highly sensitive and specific, the viral loads in the upper respiratory track peak early in the disease. These viral loads may quickly decline below the limit of detection for patients who present later in the course of infection [3]. This decline can reduce the practical applicability of this method. Serological methods can be considered a supplementary approach to fill this gap even after symptoms disappear or a lack thereof. Moreover, serological tests could also provide useful epidemiological data such as background seroprevalence, persistence of a specific class of antibodies to SARS-CoV-2 during and after the outbreak, and overall immunity status. In the past, we developed an in-house enzyme-linked immunosorbent assay (ELISA) for detecting specific IgG and IgG-isotype antibodies to SARS-CoV-2 in a Latin population that was first infected and then received an mRNA vaccine [4,5]. In this study, we standardized an in-house ELISA using SARS-CoV-2 spike RBD as the antigen to detect anti-SARS-CoV-2 IgM antibody. To standardize the assay, two panels of well-defined serum/plasma samples were used: one consisted of SARS-CoV-2-confirmed positive samples collected during the first wave of the pandemic, and the other of negative samples from healthy individuals and/or those carrying other viral/respiratory infections common in the Latin population, collected before the 2019 outbreak.

## 2. Materials and Methods

### 2.1. Antigen and Reagents

In the present study, we used a recombinant SARS-CoV-2 spike-1-RBD (Genscript No. Z03479) as an antigen, which, by SDS-PAGE analysis, exhibits a molecular weight of 30 kDa and >90% purity (Genscript, Piscayaway, NJ, USA). The assay also employed polystyrene 96-well high-binding flat-bottom microplates (Costar, Cormin Corning Cat. No. CLS3361). For the IgM ELISA, a mouse anti-human IgM-mu chain HRP conjugate (MyBiosource, Cat. No. MBS315374) was used as the secondary antibody. For the coating and buffer substrate, we used a carbonate–bicarbonate buffer with sodium azide (Sigma-Aldrich, Cat. No. C3041-100CAP) for coating and a phosphate–citrate buffer (Sigma-Aldrich, Cat. No. P4809), and 3,3′, 5, 5′-Tetramethylbenzidine (TMB) (Sigma-Aldrich Cat. No. ES001), respectively. We used the cPass SARS-CoV-2 Neutralization Antibody Detection Kit (Genscript Cat. No. L00847) customized for the variants wild type (Cat. No. Z03594), Alpha B.1.1.7 (Cat. No. Z03595), Delta B.1.617.2 (Cat. No. Z03614) and Omicron (B.1.1.529) (Cat. No. Z03730), following the manufacturer’s instructions. The clinical performance comparison was performed using the FDA-EUA-approved commercial kit SCoV-2 Detect^TM^ IgM ELISA from InBios International, Inc. (Seattle, WA, USA, Cat. No. COVE-M).

### 2.2. Plasma/Serum Samples

The study included a total of 179 samples from subjects from San Juan, Puerto Rico, of which eighty-six (86) were positive for SARS-CoV-2 and ninety-three (93) were negative for SARS-CoV-2 (Table 1). The SARS-CoV-2-positive specimens (32 serum samples and 54 plasma samples) were collected between December 2019 and March 2020. Specimens were all unidentified and kindly donated by clinical laboratories or blood banks affiliated with the University of Puerto Rico-Medical Sciences Campus (UPR-MSC) network. Since the samples were not collected specifically for this study, no personal identifiers were retained. Consequently, before the samples were received, all identifiers were removed to ensure that the information could not be traced back to the individuals. The only details retrieved from 70 out of 86 subjects were the date in which the donated specimen was collected, and the date in which the confirmatory RT-PCR for SARS-CoV-2 was performed. Hence, it was determined that 26 (30.23%) specimens had been collected between 0 to 30 days (median of 22 days) after the RT-PCR positive diagnosis; 33 (38.37%) specimens were collected between 31 to 60 days (median of 37.5 days) after the RT-PCR positive diagnosis; and 11 (12.79%) specimens were collected between 61 and 140 days (median of 84 days) following the RT-PCR positive diagnosis. The date on which the RT-PCR was carried out was not available for 16 (18.60%) of the specimens. For this study, the time elapsed between the positive RT-PCR and the date of the collection of the sample was referred to as ‘infection time’.

The negative control group samples were collected before the COVID-19 pandemic. This group comprised forty-six (46) samples from healthy subjects and forty-seven (47) from subjects carrying the most common viral or respiratory allergies encountered by the Puerto Rican population. These samples were banked at the Immunology and Molecular Parasitology Laboratory of UPR-MSC and the Virology Laboratory of UPR-MSC. The samples from subjects with viral infections were kindly donated by the Center for Disease Control and Prevention (CDC) Dengue Branch, San Juan, PR. These samples included 13 specimens from subjects with a history of respiratory allergies, 10 from subjects with a Dengue virus-IgM-positive diagnosis, 12 subjects with a RT-qPCR Influenza A/B positive diagnosis, and 6 from subjects with Respiratory Syncytial Virus (RSV)-IgM positive diagnosis. Six (6) samples presented a positive diagnosis for Mycoplasma-IgM. All samples included in the present study were kept at −80 °C and the aliquots tested were thawed since their collection.

### 2.3. In-House IgM ELISA (CovIgM-ELISA)

CovIgM-ELISA is a semiquantitative assay that uses the indirect ELISA format to detect the presence of anti-SARS-CoV-2 IgM antibody in the sera and plasma. Briefly, ninety-six-well plates were coated overnight with 100 µL/well of spike RBD recombinant protein (GenScript, USA) at a concentration of 2 μg/mL. The unbound spike RBD was removed by washing the wells three times with 350 µL/well of phosphate-buffered saline (PBS) containing 0.5% Tween-20 (PBST). Non-specific binding was blocked by adding 300 µL/well of 3% bovine serum albumin (BSA) diluted in PBST and incubated at 37 °C for 30 min. Serum or plasma samples were diluted at a ratio of 1:100 in a blocking solution and added to the wells in duplicate (100 µL/well). The blocking solution was used as a blank. Plates were washed thrice after a previous incubation period, at 37 °C for 30 min. Then, the secondary antibody, anti-human IgM-peroxidase conjugate, diluted at 1:50,000 in PBST, was added to each well (100 µL/well) and incubated at 37 °C for 30 min. The peroxidase reaction was visualized by adding the substrate solution 3,3′,5,5′-tetramethylbenzidine (TMB) and incubating at room temperature in the dark for 15 min. The reaction was stopped by adding 50 µL/well of 1 N HCl. Absorbance at 450 nm (OD_450_) was determined with a spectrophotometer. The OD_450_ of the blanks were subtracted from the OD_450_ of each sample before data analysis.

### 2.4. Plasma/Serum Equivalence and Precision Study

To assess the equivalency of the IgM determinations made for the plasma and serum, a comparison using both type of blood-derived samples, collected from the same individuals, was performed within the in-house CovIgM-ELISA. Five positive and five negative specimens were included in this study. To evaluate the precision of these determinations, these specimens were tested in duplicate three different days. Within-run and total analytical imprecision (CV) were calculated according to the Clinical and Laboratory Standards Institute (CLSI) guideline EP5-A [6].

### 2.5. Autoantibody Analysis

Human rheumatoid factor IgM was measured using an RF-IgM ELISA kit (Creative Diagnosis, Cat. No. DEIA 1697), which is a rapid test for the qualitative and semi-quantitative detection of RF-IgM-class antibody in human serum.

### 2.6. cPass Neutralization Test

Having previously demonstrated that the results provided by the surrogate viral neutralization test (cPass^TM^ GenScript sVNT, Piscataway NJ, USA) [7] correlate perfectly with the traditional PRNT [5], a cPass neutralization antibody test was used to determine the levels of neutralizing antibodies in the study population against the wild-type strain and the variants of concern (VOCs), Alpha, Delta and Omicron, which had a wide circulation and prevalence in the Puerto Rican population [8]. cPass utilizes the recombinant RBD of the SARS-CoV-2 spike protein to detect antibodies that block the RBD from binding to the human ACE2 receptor. Briefly, the specimens from negative and positive controls, as well as the standards provided by the kit were diluted 1:10 in the sample dilution buffer according to the manufacturer’s instructions and pre-incubated with RBD-HRP for 30 min at 37 °C. This allowed for the interaction and binding of specific antibodies to RBD-HRP. Following incubation, each reaction mixture was added to a 96-well capture plate coated with the human ACE-2 protein. This way, either free RBD-HRP or RBD-HRP bound to non-neutralizing antibodies could strongly interact with ACE2 being captured on the plate. RBD-HRP complexed with neutralizing antibodies remained in the supernatant and was removed in the subsequent wash step. Next, the reaction was developed with tetramethylbenzidine (TMT), giving a blue color, which turned yellow by the addition of the stop solution. The wells were read at 450 nm in an amicroplate reader. Since this was an inhibition assay, the absorbance of the sample was inversely dependent on the anti-SARS-CoV-2 neutralizing antibodies. Thus, the inhibition percentages of each sample tested was calculated as follows: percent inhibition = (1 − OD value of sample/OD value of background) × 100%. Samples with neutralization activity ≥30% indicated the presence of SARS CoV-2 RBD-interacting antibodies capable of blocking the RBD-ACE2 interaction, thus inhibiting the entrance of the virus into the cell.

### 2.7. Performance Comparison between the In-House CovIgM-ELISA and a Commercial EUA-Approved IgM-ELISA Kit

The performance of the in-house CovIgM-ELISA was compared to that of a commercially available test by using 30 samples randomly selected from the COVID-19 confirmed cohort. The collected sera were additionally tested double-blindly by two different operators using the FDA-EUA-approved commercial kit SCoV-2 Detect^TM^ IgM ELISA, which uses S1-RBD as an antigen. The serum samples for this test were processed and the results interpreted according to the manufacturer’s instructions. Each kit contained positive, negative and cut-off controls, which were provided for quality control requirements and should be tested in duplicate. According to the manufacturer’s instruction, the status of the unknown sample is determined by calculating the immunological status ratio (ISR). The ISR is calculated from the ratio of the optical density (OD) obtained with the test sample divided by the average OD of the cut-off control. Thus, if the IRS is in the range between 0.9 and 1.1, the sample status is defined as borderline and should be retested. If the ISR for a sample is ≥1.1, the sample is considered positive, and if the IRS is ≤0.9, the sample is considered negative. To complete the comparison between the in-house CovIgM-ELISA and the commercial InBios SCoV-2 Detect^TM^ IgM ELISA kit performance, a cost per sample analyzed at the laboratory level was included. The latter evaluation only considered the cost of the reagents (in-house CovIgM-ELISA) or the commercial value of the kit (InBios SCoV-2 Detect^TM^ IgM ELISA). A list with the costs in USD of all reagents used for the CovIgM-ELISA is included in Tables S1 and S2.

### 2.8. Data Analysis

To establish an accurate positive threshold that maximizes sensitivity and specificity, we generated a receiver operating characteristic (ROC) curve using the mean absorbance of all the samples tested in duplicate. The ROC curve was generated by using the EpiTools epidemiological calculator (http://epitools.ausvet.com.au) (accessed on 28 February 2024), which established the arbitrary guidelines for analyzing the area under the curve (AUC) with 95% confidence intervals for different cut-off values as follows: non-informative (AUC = 0.5); low-accurate (0.5 < AUC < 0.7; moderately accurate (0.7 < AUC < 1); and perfect-accurate (AUC = 1) [9]. To demonstrate the equivalence in the assay results paired plasma and serum specimens from the same individuals were collected and tested in our in-house-CovIgM-ELISA, and the data obtained were analyzed by a Deming regression analysis [10]. The inter-rater agreement (kappa) κ-values were applied according to the method described by Thrusfield [11] to determine the agreement between the in-house CovIgM-ELISA and the RT-qPCR, or the InBIos, Int. commercial kit, as well as between the in-house CovIgM-ELISA and the neutralization percentages determined by the surrogate cPass SARS-CoV-2 Neutralization Antibody Detection Kit. When the κ-values range between 0.01 and 0.2, the agreement is considered slight; a fair agreement is considered when κ-values range between 0.21 and 0.4; moderate agreement is considered when κ-values range between 0.61 and 0.80; and κ-values ranging between 0.81 and 1.0 is considered perfect agreement [12,13]. All statistical analyses were performed in GraphPad Prism 10.

### 2.9. Ethics Statement

None of the samples analyzed in the present study were specifically collected for this study. They were kindly donated by collaborators from local clinical laboratories or blood banks. Prior to receiving the samples, they were stripped of all identifiers so that the information could not be traced back to the individuals. Age verification was the only information gathered from most donors in which participants certified that they were >21 years old at the time the confirmatory positive RT-PCR was performed, and the serum/plasma sample was collected. The samples from the negative population were all volunteers participating in the IRB-approved clinical protocol “Molecular Basis and Epidemiology of Viral Infections Circulating in Puerto Rico” (Pro0004333), approved by the Advarra IRB on 21 April 2020.

## 3. Results

### 3.1. Receiver Operating Characteristics (ROC), OD Distribution, and the Sensitivity and Specificity of the In-House CovIgM-ELISA

**Figure 1:**
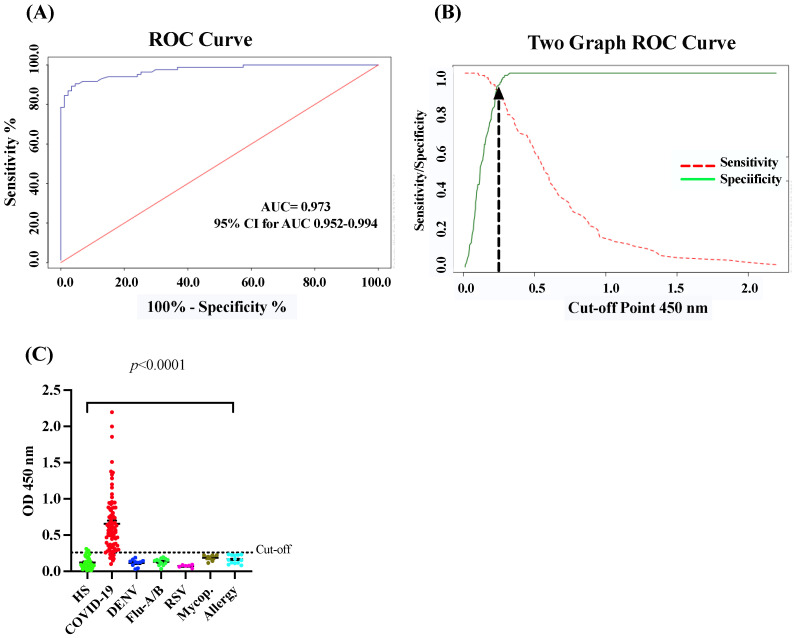
**Receiving operator characteristic (ROC) curves and OD distribution**. (**A**) ROC curve analysis for the in-house CovIgM-ELISA using S1-RBD as antigen. (**B**) two graph curves showing sensitivity and specificity for different cut-off are shown. Arrow on this graph indicates the positive cut-off value that provided maximal sensitivity (90.05%) and specificity (96.77%). (**C**) OD distribution for 86 samples from convalescent COVID-19 subjects confirmed by RT-PCR and 93 pre-pandemic samples are shown. HS: healthy subjects.

The in-house CovIgM-ELISA was standardized by measuring the background reading and using two types of controls: a pool of serum from convalescent patients as the positive control, and a pool from healthy subjects collected before the 2019 pandemic as the negative control. The optimal concentration of antigen, serum and conjugate was defined by checkerboard titration to assess reproducibility and exclude nonspecific reactions. The background of the assay was defined as the measurement from the detection system in the absence of any sample, which had to be lower than the reading of any serum ODs. Additionally, to confirm that the assay was working, positive controls and negative controls, at the optimized dilution, were used in every test. The in-house CovIgM-ELISA showed background readings that were close to zero and, as expected, all sample readings had OD values lower than the positive controls. We created ROC curves to establish the optimal positive cut-off for detecting IgM levels for SARS-CoV-2-infected samples. Moreover, to provide increased reassurance of specificity, a large panel of pre-pandemic samples collected prior to the 2019 pandemic from healthy donors, as well as from subjects previously confirmed by RT-PCR or ELISA to have viral RNA or IgG/IgM antibodies for Dengue virus (DENV), Influenza A and B, respiratory syncytial virus (RSV) and Mycoplasma, respectively, were used to further ensure specificity. ROC curve analysis indicated a mean sensitivity of 90.05% (95% CI: 82.3% to 95.10%) and a mean specificity of 96.77% (95% CI: 90.86% to 99.3%), with an AUC of 0.973 (95% CI: 0.952–0.994) (Figure 1A) and an OD_450_ positive cut-off value of 0.26, which represents a 2-fold of the mean OD of the negative samples. The cut-off represents the point in the ROC curves where the sensitivity and specificity were maximized for this set of samples (Figure 1B). Thus, when the performance of the in-house CovIgM-ELISA was compared to the RT-PCR as the standard method, the agreement between both methods was assessed as almost perfect (kappa = 0.843, 95% CI: 0.764 to 0.922) (Table 2).

To determine whether the results of the in-house CovIgM-ELISA could vary depending on the type of specimen (serum or plasma) used, paired samples from the same subject were tested and submitted for Deming regression analysis. A high equivalence between sera and plasma samples was found, which was highly significant (Figure S1). Moreover, at the comparison between duplicated runs on alternate days accomplished by different operators, CovIgM-ELISA showed an adequate consistency and low error levels, with intra-assay and inter-assay variations below 15%. The OD distribution of the validated samples is shown in a scatter plot with cut-off lines in Figure 1C. No cross-reaction was detected with sera from individuals suffering from other respiratory or viral infections. However, three samples from healthy subjects resulted positively in the in-house CovIgM-ELISA, with OD_450_ values ranging between 0.26 and 0.35.

**Figure 2:**
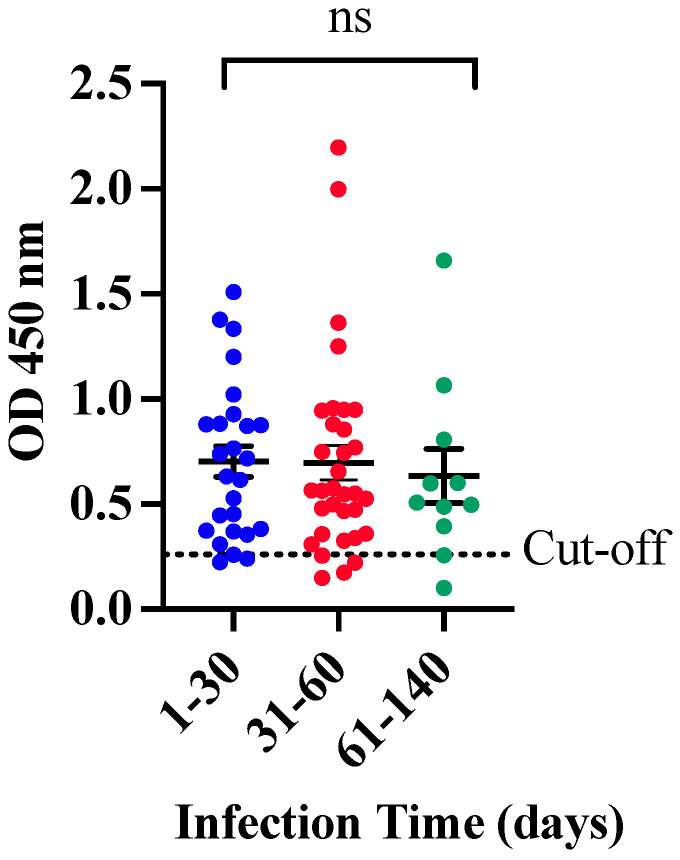
**OD distribution for IgM according to the time of infection**. Samples from convalescent COVID-19 subjects were categorized based on the time elapsed between the RT-PCR positive and the sample collection; period was termed as ‘infection time’. No significant differences (ns) were found between samples with infection time of 1 to 30 days or 31 to 60 days compared to those collected from subjects with 61 to 139 days of infection.

When the clinical performance of CovIgM-ELISA was evaluated by allotting the samples based on the estimated ‘infection time’, it was also found that 25 of 26 (96.15%) samples collected between 1 and 30 days following the RT-PCR confirmatory diagnosis were IgM-positive, whereas 30 of 33 (90.91%) specimens collected between 31 and 60 days after the RT-PCR, and 9 out of 11 (81.8%) samples from subjects with more than 60 days of infection time, after the RT-PCR, were also found to be positive to IgM. Thus, the in-house CovIgM-ELISA showed a sensitivity of 96.15% (95% CI: 80.36% to 99.90%), detecting anti-SARS-CoV-2 IgM antibody in samples from subjects with less than 30 days of infection, and a sensitivity of 93.22% (95% CI: 83.84% to 98.12%), detecting IgM in samples from subjects with up to 60 days of infection (Table 3). Paradoxically, although the sensitivity of the in-house CovIgM-ELISA decreased to the extent that the samples with longer infection times were included, the mean absorbance did not seem to decrease in the same proportion, since no significant differences were observed between the IgM levels detected in the l-to-30 days cohort, or 31 to 60 days, with respect to those collected with more than 60 days of infection (Figure 2).

To rule out that the IgM anti-SARS-CoV-2 levels detected by our in-house CovIgM-ELISA were not influenced by the rheumatoid factor (RF) as it has been previously reported [14], we proceeded to determine the presence of IgM-RF in our cohort of samples from COVID-19-convalescent subjects. This possibility cannot be ruled out, as rheumatoid arthritis (RA) is one of the most frequent autoimmune diseases in the Puerto Rican population [15], and the clinical status of the subjects used in this study is unknown. Our results demonstrated that none of the samples with anti-SARS-CoV-2 IgM antibodies were positive to IgM-RF, which confirms that the IgM levels detected in these samples were elicited against the SARS-CoV-2 and specifically detected by our in-house CovIgM-ELISA. Detailed information regarding average OD values for IgM, type of specimen, time of infection and result interpretation is provided in Table S3.

### 3.2. Clinical Performance of the In-House CovIgM-ELISA in Comparison to the SCoV-2 Detect^TM^ IgM ELISA (InBios International Inc.)

**Figure 3:**
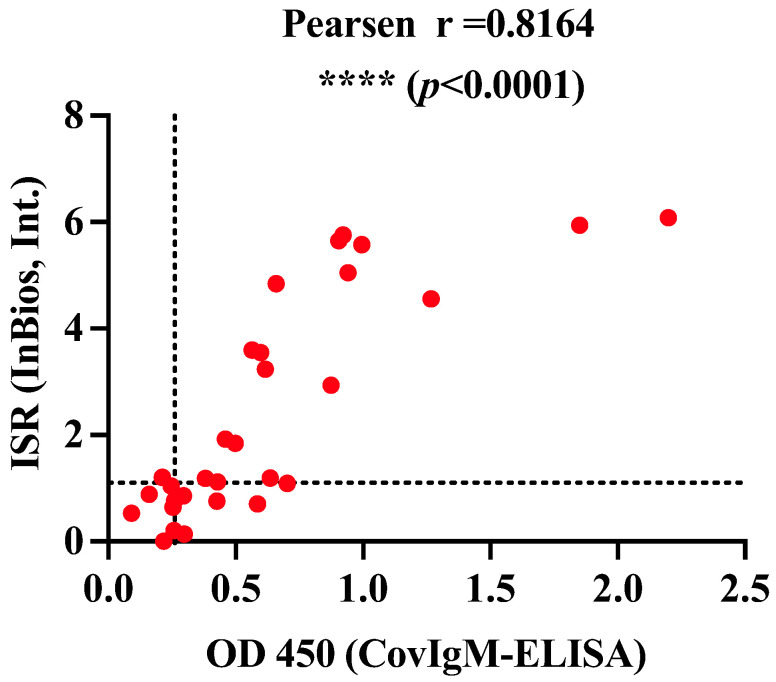
**Correlation between an in-house CovIgM-ELISA and an EUA approved commercial kit**. A subset of 30 samples from convalescent COVID-19 subjects were randomized selected and tested at double-blind by in-house CovIgM-ELISA and the EUA approved SCoV-2 Detect^TM^ IgM ELISA commercial kit (InBios, Int. LLC). The kappa assessment shown almost perfect agreement between both assays and a positive significant correlation (**** *p* < 0.0001) between the magnitude of the IgM levels reported by both assays.

The clinical performance of our in-house CovIgM-ELISA was compared with a commercial ELISA kit (SCoV-2 Detect^TM^ IgM ELISA) using a sub-cohort of 30 samples randomly selected, which were tested double-blindly by two different operators. We found that the in-house CovIgM-ELISA had substantial agreement with the SCoV-2 Detect^TM^ IgM ELISA kit (κ = 0.769, 95%CI: 0.523 to 1.00) (Table 4 and Table S4). Also, a positive and significant correlation (*p* < 0.0001) in the levels of IgM detected by both assays was found (Figure 3). The use of SCoV-2 Detect^TM^ IgM ELISA had an approximate cost of USD 7.50 per analyzed serum, whereas the in-house CovIgM-ELISA had an approximate cost of USD 2.45 per serum (Table S2), which is 3.6 times cheaper than a commercial test when labor is not considered.

### 3.3. Association between Levels of IgM Detected by the In-House CovIgM-ELISA and the Neutralizing Activity

**Figure 4:**
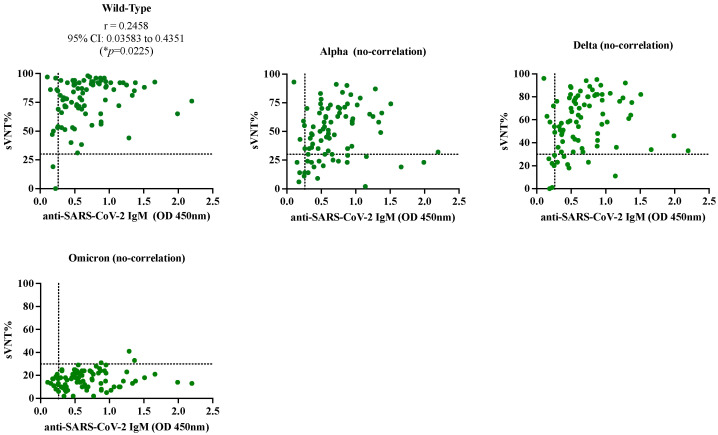
**Pearson (r) correlation between the neutralization percentages (sVNT%) and levels of anti-SARS-CoV-2 IgM**. The levels of anti-SARS-CoV-2 IgM expressed as average OD 450nm for each sample determined by the in-house CovIgM-ELISA was associated with the neutralization assay (cPass, Genscript) against the wild-type strain and the variants of concern Alpha, Delta and Omicron. Dashed lines on x-axis represent the positive cut-off value (OD > 0.26) for in-house CovIgM-ELISA and the dashed line on the y-axis represent the positive cut-off (sVNT% ≥ 30%) for the neutralization assay. There was significant correlation (* *p* = 0.02225) between sVNT% and anti-SARS-CoV-2 IgM levels for the wild-type strain.

The samples from the SARS-CoV-2-confirmed cohort were tested by a surrogate virus neutralization assay (cPass) to quantify the neutralization percentages (sVNT%) against different variant of concerns VOCs. We found that 84 of 86 samples (97.67%) had detectable sVNT% above the positive threshold (≥30%) against the wild-type (WT) strain, 64 of 86 samples (74.41%) had detectable sVNT% against the Alpha variant, 74 of 86 samples (86.05%) had detectable sVNT% against the Delta variant and only 3 of 86 samples (3.5%) samples had detectable sVNT% against Omicron. When the sVNT% were associated with the levels of IgM detected by our in-house CovIgM-ELISA, we found almost perfect agreement (Kappa = 0.95, 95% CI: 0.852 to 1.0) for the wild-type strain, slight agreement (kappa = 0.251, 95% CI: −0.014 to 0.354) for the Alpha variant and fair agreement (Kappa = 0.375, 95% CI: 0.09 to 0.658) for the Delta variant (Table 5). There was no agreement between the in-house CovIgM-ELISA and cPass for the Omicron variant. When a Pearson (r) correlation analysis was applied to determine whether the sVNT% correlated with the levels of IgM, a positive correlation (r = 0.2458, 95% CI: 0.035 to 0.4351, * *p* = 0.0225) was found for the wild-type strain and no correlation was found for the other variants (Figure 4, Table S5).

## 4. Discussion

We developed an in-house ELISA method for measuring the levels of IgM to SARS-CoV-2 in the serum/plasma of subjects infected with SARS-CoV-2 using a recombinant variant of the domain S1 from the spike protein containing the receptor binding domain (S1-RBD) as the antigen. S1 and RBD were selected as target antigens among the SARS-CoV-2 proteins because these two proteins have been reported as the most suitable for diagnostic assays. The S1 protein is highly specific whereas the RBD exhibit greater sensitivity in the detection of subjects with mild infections [16]. Other viral proteins, such as the S2 domain and nucleocapsid (N), exhibited cross-reactivity with the spike protein of MERS-CoV [16], and even more so with SARS patient sera [17]; therefore, they have been shown to be less suitable for diagnosis. In our optimized conditions, the cut-off value established over 0.26 provided high sensitivity and specificity. This positive cut-off, which represents a 2-fold of the mean OD of the negative samples, was concordant with other ELISA assays that set up their cut-off values at three standard deviations (SD) above the mean of the negatives [18].

IgM is a marker of acute infection; it can be a useful tool for detecting infection at early states (i.e., <14 days after symptoms onset) [19,20]. IgM levels can reach their peak at 15–22 days, then gradually decline, disappearing between 61 and 90 days [21,22,23], which could also reflect an antibody class switch to IgG [24]. This dynamic is consistent with the observation that the in-house CovIgM-ELISA is more sensible (96.15%) when samples are collected between 1 and 30 days of infection. Only a single sample from this group, collected one day after its RT-PCR was performed, was negative to IgM (see Table S3). Since all COVID-19 samples were donated without identifiers and no clinical data were available, it is possible to speculate that, at the time of the sampling, the adaptive immune response in this subject had not yet developed, which often occurs few days after infection.

IgM was also detected in samples collected from subjects with up to 139 days of infection. Although this was an atypical finding, it was consistent with some studies reporting the presence of anti-SARS-CoV-2 IgM antibody specific for the S1-domain in subjects with up to 90 days after the symptom onset [23], whereas other studies report that IgM antibodies specific to RBD could remain detectable 3 to 6 months after the disease onset [25]. We ruled out that these atypical IgM levels can be the result of cross-reactions with the RF or infection with other viruses because all these samples were negative to RF-IgM, and our assay did not show cross-reaction with other viruses highly endemic on the island. Moreover, the samples were also positive when tested with a commercial kit of high sensibility and specificity, which received Emergency Use Authorization (EUA) status from the U.S. Food and Drug Administration (FDA) for COVID-19. Therefore, we infer that the atypical persistence of IgM in these samples could be a characteristic of the Latin population, given that genetic background has been shown to influence response to the SARS-CoV-2 virus [26]. Alternatively, these subjects might have been reinfected with others Alpha or Delta subvariants. Although previous evidence suggests that antibodies from a primary SARS-CoV-2 infection can provide protection [27], and convalescent plasma or vaccination programs assume that humoral response can help prevent reinfection [28,29], it can still occur [30].

Despite no cross-reactions with DENV, Mycoplasma, RSV or Influenza being detected in our study, a small number of pre-pandemic samples (6.5%) from healthy donors tested positive for IgM, despite having very low OD values. We hypothesize that these false positives could be result of cross-reactions with seasonal human coronaviruses such as NL63, 229E and NL63, which have circulated within the Puerto Rican population for a long time and are responsible of a high percentage of common colds [18]. A more robust cross-reactive analysis to rule out these possibilities is needed prior to accomplish large epidemiological studies. Despite this limitation, the in-house CovIgM-ELISA has several advantages that should be highlighted. This assay is not only easy to perform, but it is also a low-cost alternative compared to the IgM-ELISA kit offered by InBios, which is one of the cheapest commercially available kits that uses S1-RBD as the target antigen. The manufacturer of ScoV-2 Detect^TM^ IgM ELISA reported a sensitivity of 96.7% when testing human serum collected between 7 and 60 days post-onset of symptoms (http://inbios.com/product/scov-2-detect-igm-elisa-kit/) (accessed on 11 September 2024). A similar sensitivity could be seen by the in-house CovIgM-ELISA when testing samples with 30 or up to 60 days of infection.

Another important observation of this study is that many IgM-positive samples exhibited remarkable neutralization percentages against the wild-type strain, and although in a smaller proportion, these samples also showed neutralizing capacity against the VOCs Alpha and Delta. This is consistent with previous studies, which demonstrated that IgM is one of the antibody classes that contribute most to SARS-CoV-2 neutralization [31]. This is attributed to the fact that these specimens were collected at the beginning of the pandemic, the time at which variants such as Alpha and Delta and their lineage, which emerged from the original Wuhan strain, were circulating in Puerto Rico [8]. Therefore, it was not unexpected that the IgM antibody elicited against the original strain could partially neutralize Alpha and Delta VOCs, because these variants accumulated a relatively small number of mutations in their genome. However, the observation that IgM antibodies became completely ineffective against Omicron was expected because this variant developed more than 50 mutations [32] that replaced Delta and other variants in circulation.

## 5. Conclusions

In conclusion, the high sensitivity, specificity and accuracy demonstrated by the in-house CovIgM-ELISA indicate that our assay could not only detect anti-SARS-CoV-2 IgM antibody, but also perform at a level comparable to that of RT-PCR and SCoV-2 Detect^TM^ IgM ELISA kits. Although RT-PCR is recognized as the standard method for detecting active SARS-CoV-2 infections, false negative results have been reported due to very low viral loads, inappropriate handling of viral samples or because the viral RNA becomes undetectable at 14 days following infection [33]. These difficulties underline the importance of developing serological tests to detect IgM, which can be detected as early as 3 days after infection. However, IgM detection alone should not be used as the only method for screening COVID-19 cases. It should also be combined with IgG detection for a more accurate and sensitive diagnostic approach. In this context, we believe that CovIgM-ELISA, along with the CovIgG-ELISA developed by our research group for detecting anti-SARS-CoV-2 IgG antibody [4,5], can be used for diagnosing suspected COVID-19 patients or for conducting large epidemiological studies in resource-limited settings, such as Puerto Rico, at a fraction of the cost, when qualified personnel are available. The in-house CovIgM-ELISA is not time-consuming; it can be completed in a maximum of 2.5 h, and 96 single samples or 48 samples in duplicate, could be tested in a single plate within the same period. Our university laboratories are well-equipped and have full-time skilled technicians. The in-house-CovIgM-ELISA is an available alternative that can make conducting COVID-19 surveillance programs at academic institutions or similar facilities with limited access to diagnostic and research resources feasible. However, despite the excellent performance showed by our in-house CovIgM-ELISA, it cannot be ignored that the validation of this assay was made with a small number of 30 samples, which is insufficient to derive clinical efficacy conclusions. Therefore, more validation studies employing a significantly larger number of samples must be performed for our in-house CovIgM-ELISA to fulfill the requirements of the Food and Drug Administration (FDA) for in vitro diagnosis (IVD) test registration.

## Figures and Tables

**Table 1:** Characteristics of COVID-19-convalescent subjects and samples from healthy subjects and those carrying other viral or respiratory infections used in the study.

Cohort 1	
Convalescent (COVID-19)	
Date of collection	04/26/2020 to 06/05/2020
Number of specimen	86 (32 sera and 54 plasma)
Day since RT-PCR confirmation test	
Range	0 to 139 days
Median	35.5 days
Number of specimens with 0 to 30 days	26
Median	22 days
Number of specimens with 31 to 60 days	33
Median	37.5 days
Number of specimens with >60 days	11
Median	84 days
Unknown	16
**Cohort 2**	
Healthy subjects	
Collection date	2012
Number included	46
Other respiratory/viral infections	
Collection date	2018–2019
Number included	47

**Table 2:** Performance for in-house CovIgM-ELISA in comparison to RT-PCR as the standard method using 86 samples from COVID-19.convalescent subjects as the ‘positive control’ group and 93 pre-pandemic samples as the ‘negative control’ group.

In-House Method		RT-PCR Standard Method Validated Samples			% Sensitivity (95% CI)	% Specificity (95% CI)	Kappa Assessment Value (95% CI)
		Positive	Negative	Total	75/86	3/93	0.843
CovIgM- ELISA	Positive	75	3	78	87.21%	96.77%	0.764 to 0.922
	Negative	11	90	101	78.27% to 93.44%	90.86% to 99.33%	Almost-Perfect Agreement
	Total	86	93	179			

**Table 3:** Sensitivity of the in-house CovIgM-ELISA according to the ‘infection time’ considering RT-PCR as the standard method.

Infection Time (IT)	N	True Positive	False Negative	Sensitivity	95% CI	Kappa Assessment (95% CI)	Interpretation
1 to 30 days	26	25	1	96.15%	80.36% to 99.90%	0.904 (0.812 to 0.996)	Almost-perfect agreement
31 to 60 days	33	30	3	90.91%	75.67% to 98.08%	0.877 (0.781 to 0.973)	
61 to 139 days	11	9	2	81.82%	48.22% to 97.72%	0.756 (0.551 to 0.961)	Substantial agreement
Sensitivity considering samples with IT between 1 and 60 days							
1 to 60 days	59	55	4	93.22%	83.54% to 98.12%	0.889 (0.813 to 0.964)	Almost-perfect agreement

IT: Time elapsed between the RT-PCR confirmatory diagnosis and the date of the specimen collection.

**Table 4:** Clinical performance of in-house CovIgM-ELISA in comparison to SCoV-2 Detect^TM^ IgM ELISA (InBios).

Commercial Method		In-House CovIgM-ELISA		
		Positive	Negative	Total
SCoV-2 Detect^TM^ IgM ELISA	Positive	19	2	21
	Negative	1	8	9
	Total	20	10	30
Kappa Assessment	0.769			
No. Observed Agreement	27 (90.00% of the observations)			
95% Confidence Interval	0.523 to 1.00			
Data Interpretation	Substantial agreement			

**Table 5:** Agreement between the results obtained by the in-house CovIgM-ELISA and the surrogate neutralization assay (cPass).

		CovIgM-ELISA			
Surrogate Neut. Assay (cPass) Wild Type		Positive	Negative	Total	Kappa assessment
	Positive	75	0	75	0.887 (almost-perfect agreement)
	Negative	9	2	11	95% CI: 0.733 to 1.0
	Total	84	2	86	No. observed agreement 84 (97.67%)
		CovIgM-ELISA			
Surrogate Neut. Assay (cPass) Alpha		Positive	Negative	Total	Kappa assessment
	Positive	60	4	64	0.251 (slight agreement)
	Negative	15	7	22	95% CI: −0.014 to 0.354
	Total	75	11	86	No. observed agreement 64 (74.42%)
		CovIgM-ELISA			
Surrogate Neut. Assay (cPass) Delta		Positive	Negative	Total	Kappa assessment
	Positive	69	5	74	0.375 (fair agreement)
	Negative	6	6	12	95% CI: 0.09 to 0.658
	Total	75	11	86	No. observed agreement 74 (86.05%)

## Data Availability

All data will be available upon requested.

## Supplementary Materials

The following supporting information can downloaded at: https://www.mdpi.com/article/10.3390/diagnostics14192209/s1, Figure S1: Deming analysis for serum/plasma equivalence and reproducibility of the in-house IgM ELISA; Table S1: In-house ConvIgM-ELISA reagents and instruments; Table S2: Bulk cost in USA dollars of all reagents used to run the in-house CovIgM-ELISA; Table S3: IgM levels measured by the in-house CovIgM-ELISA in samples from COVID-19-convalescent subjects. Cut-off >0.26; Table S4: Results obtained with the in-house CovIgM-ELISA compared to an EUA-approved commercial kit (SCoV-2 Detect^TM^ IgM ELISA); Table S5: Levels of IgM and neutralization percentages determined within samples from non-vaccinated COVID-19-convalescent subjects.
