# Supplementary material for: Anti-SARS-CoV-2 IgM Antibody Levels Measured by an In-House ELISA in a Convalescent Latin Population Persist over Time and Exhibit Neutralizing Capacity to Several Variants of Concern
Source: Diagnostics (Basel). 2024 Oct 3;14(19):2209. doi: 10.3390/diagnostics14192209 (PMC11475847; doi:10.3390/diagnostics14192209)
Supplement: Supplementary file 1 [file diagnostics-14-02209-s001.zip › diagnostics-3183579-supplementary.pdf]

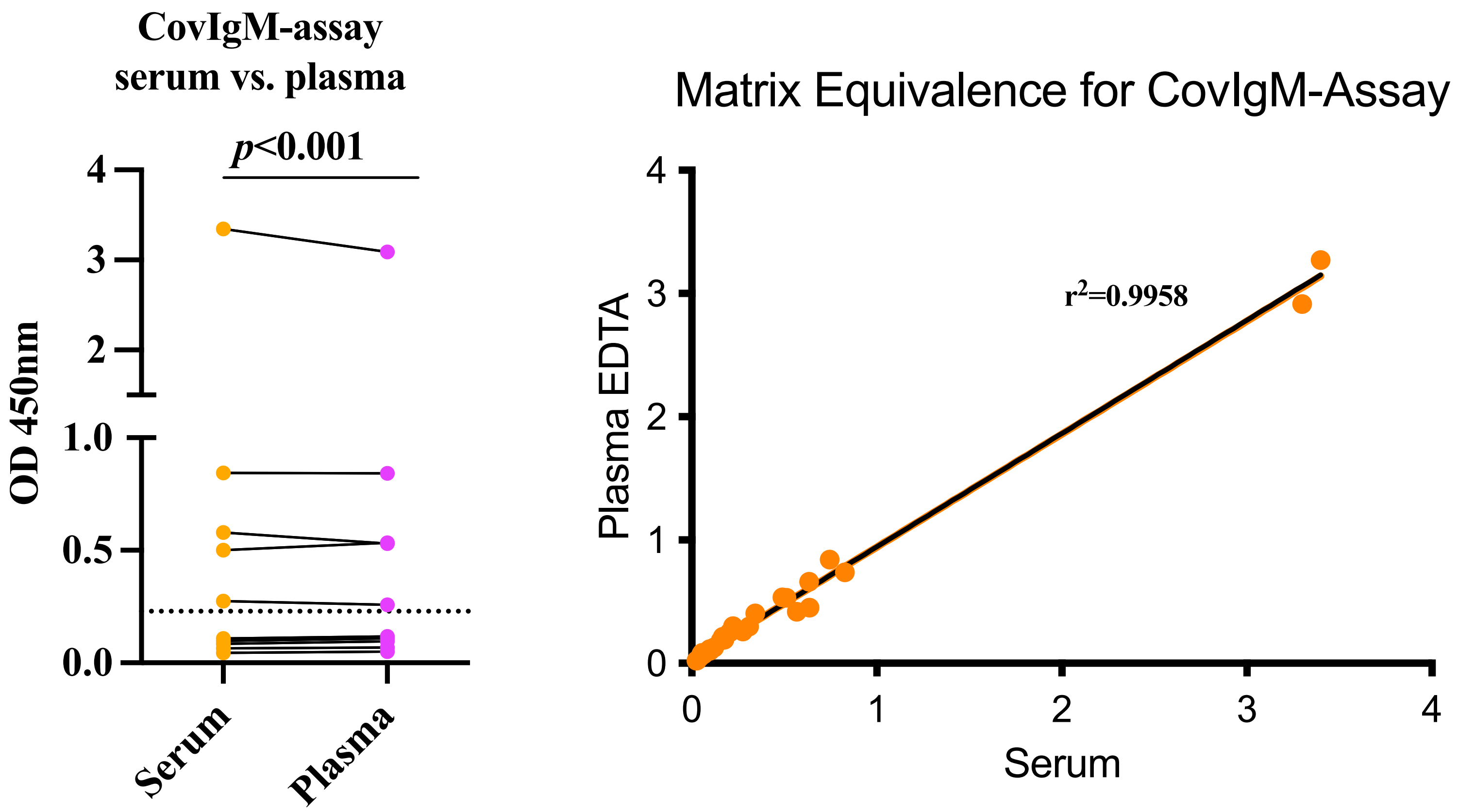

Figure-S1. Equivalence study made with the In-house CovIgM-ELISA. To determine whether if the IgM determinations could vary depending on the type of specimen (serum or plasma) used, paired samples from the same subject were tested by in house CovIgM-ELISA and the results were submitted to a Deming analysis. There was a high ecorrelation (r^2=9.9958) between the results retrieved fron sera and plasma samples and the equivalence was highly significant ($p<0.001$).

Table-S1. In house-CovIgM-ELISA reagents and instruments.

List of all reagents and instruments (brand and source) used to run the assay.

Reagents and Kits

1. Recombinant Spike-S1-RBD His Tag: GenScript Cat. # Z03479, stock |Lot P50462003| [1.2 mg/ml], Piscataway, NJ, USA
2. 96-well plate: Pkg of 100 plates High-bind, clear, flat-bottomed, polystyrene (Corning, Cat. # CLS3361)
3. PBS: 0.1M phosphate-buffered saline pH 7.0, (Sigma-Aldrich, Cat. # TMS-012)
4. Tween-20: Sigma-Aldrich (Cat. # P1379)
5. BSA: Bovine serum albumin Fraction V (Sigma-Aldrich, Cat. # 10735086001)
6. Coating buffer: 100 Capsules of carbonate-bicarbonate buffer pH 8.0 (Sigma-Aldrich Cat. # C3041-100CAP
7. Goat anti-human IgM-(mu) polyclonal antibody HRP labelled: MyBiosource (2mg) Cat. # MBS315374
8. TMB Substrate: (3,3', 5, 5'-Tetramethylbenzidine) Sigma-Aldrich Cat. # ES001
9. HCl: Hydrochloric acid 36.5-38.0%, Sigma-Aldrich, Cat. # H1758

Instruments

Microcentrifuge: Eppendorf (Model 5415R)

Freezer -80°C: Sanyo Ultra Low (Model MDF-U32V)

Refrigerator 4°C: Thermo Scientific (Model GTTSG45RPLA)

Microplate Reader: Thermo Scientific Multiskan FC (Model 51119000)

Incubator (37°C): Barnstead Lab-Line (Model 150)

Table S2. Cost of in-house ELISA reagents. Bulk cost in USA dollars of all reagents used to run CovIgM-ELISA

Article	Manufacturer	Catalog Number	Unit Price (US dollars)	Units	Total
**Protein/Antibody**					
Recombinant Spike-S1-RBD His Tagged-1mg	GenScript	Z03479	$2,100.00	1	$2,100.00
Goat anti-human IgM-(mu) polyclonal antibody HRP labelled (2mg)	MyBiosource	MBS315374	$295.00	1	$295.00
**Reagents**					
BSA-Fraction V 98.5% (100g)	Roche	10735086001	$355.00	1	$355.00
Tween-20 (100ml)	Sigma-Aldrich	P1379	$38.00	1	$38.00
PBS 1X (1L)	Sigma-Aldrich	TMS-012	$36.10	10	$360.10
TMB Substrate	Sigma-Aldrich	ES001	$140.00	1	$140.00
Carbonate-bicarbonate buffer pH 8.0-100 Capsules	Sigma-Aldrich	C3041-100CAP	$197.00	1	$197.00
Hydrochloric acid (HCl)-100ml	Sigma-Aldrich	H1758	$83.00	1	$83.00
**Plastics**					
96-well plates (pkg 40)	Millipore-Sigma	MSEHNFX40	$201.00	1	$201.00
Microtubes-1.5ml (pkg 2,500 tubes)	Axygen-Corning	AXYMCT150CS	$201.00	2	$402.00
Tubes-15ml (pkg 500 tubes)	Corning	CLS430791	$332.00	4	$1,328.00
Tubes-50ml (pkg 500 tubes)	Corning	CLS430829	$456.00	4	$1,824.00
Tips-P10 (pkg of 10 racks x 96 tips)	Eppendorf	0030078500	$214.00	3	$606.00

Tips-P200 (pkg of 10 racks x 96 tips)	Eppendorf	0030078551	$202.00	3	$606.00
Tips-P1000 (pkg of 10 racks x 96 tips)	Eppendorf	0030078578	$202.00	3	$606.00
			**Sub-Total**		$9,141.1
			**Shipping Cost**		$275.00
			**Total**		$9,416.10
**With 40 plates we can test 3840 single samples (1920 samples in duplicate)**					
**Cost (USD) per sample**					**$2.45**

**SCoV-2 Detect™ IgM ELISA kit, InBios International. Cat. # COVE-M**		
**Catalog Prize**	**Shipping Cost**	**Total**
**$683.00**	**$39.00**	**$722.00**
**The kit includes a single 96-well plate, which permit the testing of 96 single samples.**		
**Cost (USD) per sample**		**$7.52**

Table S3. IgM levels measured by the in-house CovIgM-ELISA in samples from COVID-19 convalescent subjects. Cut-off >0.26

No.	ID	Specimen	Time of Infection	Average OD	Result Interpretation
1	BBMC102	Serum	Unknown	0.489	Positive
2	BBMC103	Serum	Unknown	0.840	Positive
3	BBMC104	Serum	Unknown	0.601	Positive
4	BBMC105	Serum	35	0.949	Positive
5	BBMC106	Serum	35	0.309	Positive
6	BBMC132	Serum	32	0.856	Positive
7	BBMC133	Serum	37	1.363	Positive
8	BBMC134	Serum	32	0.467	Positive
9	BBMC135	Serum	37	0.546	Positive
10	BBMC136	Serum	38	0.221	Negative
11	BBMC137	Serum	34	0.552	Positive
12	HAM 143	Serum	1	0.871	Positive
13	BBMC 147	Serum	35	1.251	Positive
14	BBMC 148	Serum	39	0.565	Positive
15	LMH 151	Serum	48	0.498	Positive
16	BSCM 152	Serum	45	0.525	Positive
17	LML 153	Plasma	48	0.769	Positive
18	BBSM 155	Plasma	20	0.447	Positive
19	BBSM 156	Plasma	47	0.325	Positive
20	BBSM 157	Plasma	27	0.453	Positive
21	BBSM 158	Plasma	20	0.633	Positive
22	BBSM 159	Plasma	26	0.929	Positive
23	BBSM 160	Plasma	31	0.174	Negative
24	BBSM 161	Plasma	22	0.738	Positive
25	BBSM 162	Plasma	48	0.481	Positive
26	BBSM 163	Plasma	27	0.309	Positive
27	BBSM 164	Plasma	29	0.369	Positive
28	BBSM 165	Plasma	30	0.260	Negative
29	BBSM 166	Plasma	44	0.749	Positive
30	BBSM 167	Plasma	22	0.882	Positive
31	BBSM 170	Plasma	48	0.957	Positive
32	BBSM 171	Plasma	41	0.944	Positive
33	BBSM 173	Plasma	33	0.745	Positive
34	BBSM 176	Plasma	42	0.949	Positive
35	BBSM 177	Plasma	36	0.575	Positive
36	BBSM 178	Plasma	36	0.360	Positive
37	BBSM 179	Plasma	14	0.374	Positive
38	BBSM 180	Plasma	13	0.383	Positive
39	BBSM 181	Plasma	48	0.656	Positive
40	BBSM 182	Plasma	38	0.880	Positive
41	BBSM 184	Serum	37	1.999	Positive

42	BSSM 192	Plasma	Unknown	0.251	Negative
43	BBMC 193	Plasma	54	0.357	Positive
44	BBMC 195	Serum	61	0.807	Positive
45	BBSM 199	Serum	61	0.258	Negative
46	BSSM 201	Plasma	29	0.223	Negative
47	BBSM 207	Plasma	54	0.148	Negative
48	BBSM 209	Plasma	34	0.339	Positive
49	BBSM 210	Plasma	18	1.202	Positive
50	BBSM 214	Serum	96	1.066	Positive
51	BBSM 218	Plasma	Unknown	1.139	Positive
52	BBSM 220	Plasma	69	0.488	Positive
53	BSSM 229	Plasma	35	0.255	Negative
54	BBSM 231	Serum	28	0.765	Positive
55	UPR 247	Plasma	Unknown	0.185	Negative
56	LV 260	Serum	1	1.334	Positive
57	LV 261	Serum	1	0.717	Positive
58	LV 262	Plasma	1	0.241	Negative
59	BSSM 275	Plasma	84	0.102	Negative
60	BBSM 284	Plasma	84	0.508	Positive
61	BBSM 288	Serum	116	0.602	Positive
62	LCTC 300	Plasma	25	0.881	Positive
63	LCTC 312	Plasma	14	0.616	Positive
64	BBSM 318	Plasma	15	1.022	Positive
65	BBSM 322	Plasma	33	0.473	Positive
66	LCTC 367	Serum	Unknown	1.155	Positive
67	LCTC 374	Serum	Unknown	1.285	Positive
68	LCTC 375	Serum	Unknown	0.689	Positive
69	LCTC 376	Serum	Unknown	0.299	Positive
70	LCTC 377	Serum	Unknown	0.191	Negative
71	LCTC 381	Plasma	36	2.196	Positive
72	BSSM 382	Serum	Unknown	0.639	Positive
73	LCTC 384	Plasma	Unknown	0.603	Positive
74	BBSM 386	Plasma	42	0.564	Positive
75	BBSM 390	Plasma	26	1.378	Positive
76	BBSM 391	Plasma	68	0.394	Positive
77	BBSM 393	Plasma	25	0.876	Positive
78	BBSM 397	Plasma	20	0.356	Positive
79	BBSM 403	Plasma	23	1.509	Positive
80	BBSM 409	Plasma	116	0.598	Positive
81	BBSM 412	Plasma	84	1.66	Positive
82	BBSM 417	Plasma	29	0.529	Positive
83	BBSM 427	Plasma	139	0.496	Positive
84	LCTC 451	Serum	Unknown	0.291	Positive
85	LCTC 457	Plasma	Unknown	0.670	Positive
86	LCTC 471	Serum	Unknown	0.541	Positive

Table S4. Results obtained with the in-house CovIgM-ELISA compared to an EUA Approved Commercial kit (SCoV-2 Detect™ IgM ELISA).

			**CovIgM-ELISA (Cut-off >0.26)**		**SCoV-2 Detect™ IgM ELISA (IRS >1.1)**	
**No.**	**ID**	**Infection time (days)**	**Average OD ± SD**	**Result**	**Average IRS ± SD**	**Result**
**1**	LMH 151	48	0.459 + 0.061	Positive	1.923 + 0.026	Positive
**2**	BBSM 155	20	0.425 + 0.066	Positive	0.754 + 0.023	Negative
**3**	BBSM 158	20	0.598 + 0.076	Positive	3.547 + 0.129	Positive
**4**	BBSM 159	26	0.941 + 0.123	Positive	5.050 + 0.060	Positive
**5**	BBSM 160	31	0.160 + 0.030	Negative	0.883 + 0.006	Negative
**6**	BBSM 163	27	0.295 + 0.053	Positive	0.855 + 0.021	Negative
**7**	BBSM 165	30	0.260 + 0.025	Negative	0.769 + 0.007	Negative
**8**	BBSM 166	44	0.702 + 0.202	Positive	1.192 + 0.018	Positive
**9**	BBSM 199	61	0.258 + 0.054	Negative	0.139 + 0.005	Negative
**10**	BBSM 170	48	0.905 + 0.386	Positive	5.652 + 0.138	Positive
**11**	BBSM 171	41	0.922 + 0.089	Positive	5.760 + 0.071	Positive
**12**	BBSM 192	Unknown	0.258 + 0.030	Negative	0.203 + 0.012	Negative
**13**	BBSM 201	29	0.218 + 0.095	Negative	0.002 + 0.001	Negative
**14**	BBSM 207	54	0.246 + 0.072	Negative	1.042 + 0.034	Negative
**15**	BBSM 229	35	0.254 + 0.068	Negative	0.640 + 0.010	Negative
**16**	LV 260	0	0.659 + 0.154	Positive	4.556 + 0.083	Positive
**17**	LV 261	0	1.268 + 0.212	Positive	4.843 + 0.008	Positive
**18**	LV 262	0	0.212 + 0.017	Negative	1.206 + 0.167	Positive
**19**	BBSM 275	84	0.091 + 0.014	Negative	0.533 + 0.010	Negative
**20**	BBSM 284	84	0.498 + 0.047	Positive	1.842 + 0.072	Positive
**21**	BBSM 288	116	0.565 + 0.149	Positive	3.599 + 0.016	Positive
**22**	LCTC 300	25	0.874 + 0.097	Positive	2.940 + 0.061	Positive
**23**	LCTC 312	14	0.616 + 0.105	Positive	3.235 + 0.001	Positive
**24**	BBSM 318	15	0.995 + 0.221	Positive	5.577 + 0.047	Positive
**25**	UPR 381	36	2.199 + 0.649	Positive	6.081 + 0.030	Positive
**26**	LCTC 378	Unknown	0.820 + 0.036	Positive	1.857 + 0.012	Positive
**27**	BBSM 393	25	0.289 + 0.008	Positive	1.019 + 0.058	Positive
**28**	BBSM 412	84	1.860 + 0.693	Positive	5.943 + 0.200	Positive
**29**	BBSM 427	139	0.428 + 0.038	Positive	1.114 + 0.011	Positive
**30**	BBSM 471	Unknown	0.381 + 0.112	Positive	1.181 + 0.001	Positive

Table S5. Levels of IgM and neutralization percentages determined within samples from non-vaccinated, COVID-19 convalescent subjects.						
		CovIgM-ELISA	sVNT% (cPass)			
**No.**	**Sample ID**	**Average OD**	**Wild type**	**Alpha**	**Delta**	**Omicron**
1	BBMC102	0.489	92	74	82	25
2	BBMC103	0.840	92	71	81	22
3	BBMC104	0.601	69	33	42	24
4	BBMC105	0.949	88	59	82	29
5	BBMC106	0.309	53	14	36	25
6	BBMC132	0.856	91	63	82	26
7	BBMC133	1.363	85	49	64	33
8	BBMC134	0.467	53	24	18	21
9	BBMC135	0.546	87	54	77	29
10	BBMC136	0.221	0	-8	22	8
11	BBMC137	0.552	70	30	43	18
12	HAM143	0.871	96	90	95	24
13	BBMC147	1.251	90	63	79	23
14	BBMC148	0.565	92	63	79	22
15	LMH151	0.498	94	78	88	25
16	BSCM152	0.525	72	52	45	24
17	LML153	0.769	93	70	89	2
18	BBSM155	0.447	40	9	21	17
19	BBSM156	0.325	77	35	54	9
20	BBSM157	0.453	75	35	58	18
21	BBSM158	0.633	94	73	85	24
22	BBSM159	0.929	96	82	90	24
23	BBSM160	0.174	47	6	26	17
24	BBSM161	0.738	91	63	80	23
25	BBSM162	0.481	84	50	59	20
26	BBSM163	0.309	66	30	36	24
27	BBSM164	0.369	78	47	49	6
28	BBSM165	0.260	69	35	49	6
29	BBSM166	0.749	55	24	23	16
30	BBSM167	0.882	58	29	48	8
31	BBSM170	0.957	89	61	65	5
32	BBSM171	0.944	77	37	56	15
33	BBSM173	0.745	93	71	80	17
34	BBSM176	0.949	93	57	77	22
35	BBSM177	0.575	79	45	58	14
36	BBSM178	0.360	71	36	47	18
37	BBSM179	0.374	71	39	41	10
38	BBSM180	0.383	78	54	51	16
39	BBSM181	0.656	65	30	35	10
40	BBSM182	0.880	94	74	82	31
41	BBSM184	1.990	65	23	46	14

42	BSSM192	0.251	53	11	20	12
43	BBMC193	0.357	51.3	23	32	7
44	BBMC195	0.807	96	84	86	28
45	BBSM199	0.258	54	14	29	13
46	BBSM201	0.223	96	-2	1	19
47	BBSM207	0.148	86	23	63	13
48	BBSM209	0.339	79	44	51	2
49	BBSM210	1.202	92	65	76	15
50	BBSM214	1.066	86	79	83	10
51	LCTC218	1.139	72	2	11	10
52	BBMC220	0.488	89.9	83	89	20
53	BBSM229	0.255	85	55	54	17
54	BBSM231	0.765	90	66	72	21
55	UPR247	0.185	19	14	0	17
56	LV260	1.334	81	58	61	13
57	LV261	0.717	97	91	94	24
58	LV262	0.241	86	59	72	21
59	BBSM275	0.102	97	93	96	14
60	BBSM284	0.508	91	70	67	18
61	BSSM288	0.602	77	51	64	20
62	LCTC300	0.881	56	35	42	7
63	LCTC312	0.616	83	44	70	15
64	BBSM318	1.022	92	73	58	7
65	BBSM322	0.473	94	66	79	2
66	LCTC367	1.155	92	28	36	10
67	LCTC374	1.285	44	87	92	41
68	LCTC375	0.689	98	47	73	10
69	LCTC376	0.299	94	24	23	10
70	LCTC377	0.191	50	43	58	11
71	UPR381	2.196	76	32	33	13
72	BBSM382	0.639	72	58	62	12
73	LCTC384	0.603	87	48	54	10
74	BBSM386	0.564	71.4	62	74	21
75	BBSM390	1.378	92	66	75	15
76	BBSM391	0.394	92	19	28	7
77	BBSM393	0.876	65	23	37	13
78	BBSM397	0.356	72	48	57	12
79	BBSM403	1.509	88	74	82	18
80	BBSM409	0.598	38.3	70	81	20
81	BBSM412	1.660	92.6	19	34	21
82	BBSM417	0.529	73	20	32	14
83	BBSM427	0.496	51.8	42	70	21
84	LCTC451	0.291	81	70	76	18
85	LCTC457	0.670	86	25	32	7
86	LCTC471	0.541	31	58	76	15

Kappa Analysis between: CovIgM-ELISA and cPass (Wild-type)

Both techniques agree consider positive: 75
Both techniques agree consider negative: 2
Only cPass consider positive: 9
Only CovIgM-ELISA consider positive: 0

Kappa: 0.887 (almost perfect agreement)
95% CI: 0.733 to 1.0
No. Observed agreement: 84 (97.67% of the observations)

Kappa Analysis between: CovIgM-ELISA and cPass (Alpha)

Both techniques agree consider positive: 60
Both techniques agree consider negative: 7
Only cPass consider positive: 4
Only CovIgM-ELISA consider positive: 15

Kappa: 0.251 (Slight agreement)
95% CI: -0.014 to 0.354
No. Observed agreement 64 (74.42% observations)

Kappa Analysis between: CovIgM-ELISA and cPass (Delta)

Both techniques agree consider positive: 69
Both techniques agree consider negative: 6
Only cPass consider positive: 5
Only CovIgM-ELISA consider positive: 6

Kappa: 0.375 (Fair agreement)
95% CI: 0.09 to 0.658
No. Observed agreement 74 (86.05%)
